# Tissue expression profiles and transcriptional regulation of elongase of very long chain fatty acid 6 in bovine mammary epithelial cells

**DOI:** 10.1371/journal.pone.0175777

**Published:** 2017-04-17

**Authors:** Si Chen, Hua He, Xiaolin Liu

**Affiliations:** 1 Shaanxi Key Laboratory of Molecular Biology for Agriculture, College of Animal Science and Technology, Northwest A&F University, Yangling, Shaanxi, China; 2 College of Veterinary Medicine, Northwest A&F University, Yangling, Shaanxi, China; Hokkaido Daigaku, JAPAN

## Abstract

In mammals, very long chain fatty acids (VLCFAs) perform pleiotropic roles in a wide range of biological processes, such as cell membrane formation, cell signal transduction, and endocrine regulation. Beef and milk are abundant of palmitic acid which can be further elongated into stearic acid for synthesizing VLCFAs. Elongase of very long chain fatty acid 6 (ELOVL6) is a rate-limiting enzyme for converting palmitic acid to stearic acid. Consequently, investigating the tissue expression patterns and transcriptional regulation of bovine *ELOVL6* can provide new insights into improving the composition of beneficial fats in cattle and expanding the knowledge of transcriptional regulation mechanism among domestic animals. In the current study, we found that bovine *ELOVL6* expressed ubiquitously. Dual-luciferase reporter assay identified that the core promoter region (-130/-41 bp) was located in the second CpG island. In addition, the deletion mutation of binding sites demonstrated that sterol regulatory element binding transcription factor 1 (SREBF1) and specific protein 1 (SP1) both were able to stimulate bovine *ELOVL6* promoter activity independently, while resulting the similar effect. To confirm these findings, further RNA interference assays were executed in bovine mammary epithelial cells (BMECs). In summary, these data suggest that bovine *ELOVL6* expressed ubiquitously and is activated by SREBF1 and SP1, via two binding sites present in the *ELOVL6* promoter region between -130 bp to -41bp.

## Introduction

In vertebrates, increasing evidence has shown that very long chain fatty acids (VLCFAs) which are the fatty acids of 20 carbon or more in length perform a vital role in maintaining global metabolic homeostasis and normal physiological function [1]. Among VLCFAs, the polyunsaturated fatty acids (PUFAs) arachidonic acid and docosahexaenoic acid regulate several processes within the brain, including neurotransmission, cell survival and neuroinflammation [2]. PUFAs also participate in lipid metabolism by directly interacting with the lipid-sensing transcription factors (TFs) [3]. People benefit greatly from livestock products, such as beef and milk, which provide nutrition containing high-quality protein, and low-level fat with a desirable VLCFAs profile. Oleic acid (18:1n-9, 35.70%) which belongs to VLCFAs is the most abundant fatty acid in beef, followed by palmitic acid (16:0, 31.07%) [4]. With the help of mid-infrared predictions tools, milk was detected to possess more palmitic acid (33.44%) than oleic acid (17.31%) [5]. Lately researches showed that diets high in oleic acid improved the health condition for individuals through the effect on reducing central obesity and cardiovascular disease risk [6, 7].

In mammals, fatty acids with a chain length of up to 16 carbons are synthesized by fatty acid synthase(FASN), as well as are gained from diet. These short chain fatty acids (SCFAs) are further elongated and desaturated into VLCFAs [8]. The elongation of SCFAs is proceeded by a four-step biochemical cycle. In the elongation cycle, the first rate-limiting (condensation) step was catalyzed by a group of endoplasmic reticulum (ER) membrane-bound enzymes, termed ELOVL (Elongase of very long chain fatty acids) [9]. To date, seven distinct isoforms of ELONGASE family have been identified, which were designated from ELOVL1 to ELOVL7, reside in murine and human [10]. Each ELOVL protein exhibits a characteristic substrate specificity [11].

C18 fatty acids has been proved to be the precursor for the synthesis of VLCFAs [12]. ELOVL6 (LCE/FACE) is essential for synthesizing C18 fatty acids, owning to its specific activity to convert C16 saturated and monounsaturated fatty acids into C18 fatty acids [13]. Previous study indicated that *ELOVL6* was ubiquitously expressed, especially in tissues with high lipid content such as brown/white adipose tissue, liver and brain in mouse [14]. The function researches of *ELOVL6* demonstrated that the deficiency of *ELOVL6* in mouse protected against metabolic diseases such as insulin resistance [15], nonalcoholic steatohepatitis [16]. Meanwhile, the overexpress of mouse *ELOVL6* induced cancer diseases, included breast cancer [17], cystic fibrosis [18], pulmonary fibrosis [19] and lung squamous cell carcinoma [20]. *ELOVL6* also regulates thermogenic capacity in brown adipose tissue [21]. Consequently, many researches poured attentions into the transcriptional regulation of *ELOVL6*.

Recent attempts utilizing advanced molecular biological techniques have provided some novel insights of the transcriptional regulation of *ELOVL6*. In general, sequence-specific transcription factors have been grouped into two categories: proximal promoter factors, and enhancer binding factors. Direct evidence indicated that hepatic expression of *ELOVL6* in mouse was regulated by SREBF1 via SREBF1 binding sites (SRE) present in the *ELOVL6* promoter. Further ChIP assay showed that the proximal SRE-1 binding site on the promoter of *ELOVL6* had higher affinity to SREBF1 than the distal one [22]. Meanwhile, a recent study suggested that human carbohydrate response element binding protein (ChREBP) and SREBF1 synergistically stimulated *ELOVL6* promoter activity in HepG2 cell lines [23]. It has been demonstrated that a portion of transcriptional regulation which was activated by SREBPs requires cooperation with other DNA binding transcription factors such as SP1, NF-Y, and CREB as well as with coactivators [24].The SREBF family is essential to the regulation of milk lipogenic genes expression, including acetyl-CoA carboxylase (ACC), fatty acid synthetase (FAS), stearoyl-CoA desaturase (SCD), mechanistic target of rapamycin (mTOR), desaturation fatty acid binding protein 3 (FABP3) and peroxisome proliferator activated receptor γ (PPARγ) [25]. Additionally, among the transcription factors involved in lipid metabolism of beef cattle, PPARs and SREBFs stand out [26].

To address the question of whether the transcriptional pattern of *ELOVL6* in bovine is conserved, we determined the tissue expression profile of bovine *ELOVL6* in nine different tissues by quantitative real-time PCR (qPCR). In order to identify and narrow down the core promoter region of bovine *ELOVL6*, we constructed six dual-luciferase reporter plasmids harboring various 5’ flanking truncations and detected their promoter activities by dual-luciferase reporter assays. Seven transcription factor binding sites (TFBS) were predicated in the core promoter region of bovine *ELOVL6*. Subsequently, the dominant transcription factors were verified by site-directed deletion mutation and RNA interference assays. Our results suggest that bovine ELOVL6 expressed ubiquitously and is activated by SREBF1 and SP1, via two binding sites present in the ELOVL6 promoter region between -130 bp to -41bp.

## Materials and methods

### Ethics statement

All animal procedures were carried out in accordance with the Regulations for the Administration of Affairs Concerning Experimental Animals (Ministry of Science and Technology, China, 2004) and were approved by the Institutional Animal Care and Use Committee at the Northwest A&F University (Protocol NWAFAC1117). Cattles were raised under free food intake and humanely slaughtered in the Shannxi Kingbull Animal Husbandry Company, Ltd (Baoji, Shaanxi, China).

### Quantitative real-time PCR (qPCR)

The tissues were collected from three 2 year-old male Qinchuan cattles, including heart, liver, spleen, lung, kidney, intestine, stomach, skeletal muscle and abdominal fat. The relative quantification was carried out against the quantification cycle (Cq) value of *ELOVL6* in spleen tissue. Total RNA was extracted using Trizol reagent (Invitrogen, USA) and quantified by the Nanodrop 2000 spectrophotometry (Thermo Fisher Scientific, USA). cDNA was subsequently synthesized by using the All-in-one RT MasterMix (ABM, USA). The quantitative real-time PCR (qPCR) reactions were carried out in a CFX96 Real-Time PCR Detection System (Bio-Rad, USA) employing the SYBR^®^ Premix Ex Taq II (Takara, China). The Cq values were normalized to reference gene (GAPDH) run on the same plate. The primers which amplified the transcripts were listed in S1 Table. All the experiments were performed in triplicates.

### Dual-luciferase reporter assays

The 5’-flanking region of bovine ELOVL6 promoter was amplified and was identical to the GenBank database (Accession no. AC_000163). In order to determine the core region of bovine *ELOVL6* promoter, multiple dual-luciferase reporter plasmids containing unidirectional truncations (from 5’ to 3’) were constructed. The primers were listed in S2 Table. After double digestions with *Mlu*I and *Xho*I, the fragments were cloned into pGL3-basic vector, respectively. The resulting constructs were designated as pGL3-F2, pGL3-F3, pGL3-F4, pGL3-F5, pGL3-F6 and pGL3-F7. Deletion mutation constructs were subsequently generated by overlapping extension methods using pGL3-F5 as template. All constructs were sequenced in both directions (Invitrogen, USA). The primers were listed in S3 Table. Constructed plasmids were then co-transfected with pRL-TK plasmid into 3T3-L1 and 293A cell lines for the dual-luciferase reporter assay.

### RNA interference

All siRNAs were designed and synthesized according to the online prediction program at http://rnaidesigner.thermofisher.com/rnaiexpress/. *SREBF1*-siRNA sequences were as following, sence: 5'- UCUUCCAUCAAUGACAAGATT-3', anti-sence: 5'- UCUUGUCAUUGAUGGAAGATT-3'. *SP1*-siRNA sequences were as following, sence: 5'-GCCAAUAGCUACUCAACAATT-3', anti-sence: 5'-UUGUUGAGUAGCUAUUGGCTT-3'. Control-siRNA sequences were as following, sence: 5'- UUCUCCGAACGUGUCACGUTT-3', anti-sence: 5'- ACGUGACACGUUCGGAGAATT-3'. siRNAs were then transfected into BMECs following the manual of Lipofectamine™ 2000 (Invitrogen, USA). The expressions were measured by qPCR described above.

### Cell culture and transfection

3T3-L1 (CL-173, ATCC) and 293A (R70507, Invitrogen) cell lines were maintained in Dulbecco’s modified eagle’s medium (DMEM) (Hyclone, GE, USA) which was supplemented with 10% fetal bovine serum (FBS) (Gibco, Invitrogen, USA) in 5% CO2 and 100% humidity at 37℃ and passaged using standard cell culture techniques. Before transfection, cells were plated at a density of 1.4×10^5^ cells per well in 48-well plates and incubated for 12 hours until they reached 80–90% confluent. Plasmids described above were transfected using Lipofectamine 2000 (Invitrogen, USA) according to the manufacturer’s instructions. After 48 hours transfection, cells were washed with 1× PBS and lysed with 1× passive lysis buffer for 15 min. Dual-luciferase reporter assay was carried out by using Varioskan Flash instrument (Thermo Fisher Scientific, USA). The level of firefly luciferase activity was normalized to renilla luciferase activity. In RNA interference assays, BMECs were cultured in 1640 medium (Hyclone, GE, USA) which was supplemented with 10% fetal bovine serum (FBS) (Gibco, Invitrogen, USA) in 5% CO2 and 100% humidity at 37℃ and passaged using standard cell culture techniques. Before transfection, cells were plated at a density of 7.5×10^5^ cells per well in 48-well plates and incubated for 12 hours until they reached 70–80% confluent. siRNAs (20 umol/L) were transfected as 5 pmol mixed with 0.25 ul Lipofectamine 2000 per well. After 24 hours and 48 hours, samples of siRNA treatment were collected.

### Bioinformatics analyses

The bovine *ELOVL6* promoter was analyzed using a promoter prediction program at http://www.cbs.dtu.dk/services/Promoter/. The transcription factor binding sites were predicted by using an online prediction server at http://www.biobase-international.com/product/transcription-factor-binding-sites. The methylation CpG island of bovine *ELOVL6* promoter was analyzed at http://www.urogene.org/cgi-bin/methprimer/methprimer.cgi. For the phylogenetic analysis, other promoter sequences from different mammalian species were aligned with bovine *ELOVL6* promoter by the Clustal X 2.1 program. A phylogenetic tree was constructed by using the MEGA program (version 6.0) with the neighbor-joining method. Bootstrap values were obtained from 1000 repetitions and listed as percentages at the nodes. The core regions of promoter sequences from different mammalian species were aligned and visualized by DNAman software (version 7.0).

### Statistical analyses

SPSS 19.0 (IBM, Armonk, NY, USA) software performed all statistical analyses. The relative expression quantification was evaluated by the algorithm of 2^-ΔΔCT^ method. The results of each independent samples were normalized to the reference gene (GAPDH) run on the same plate. A one-way ANOVA test was conducted to determine the significant level. Mean values were compared by the LSD post-test. The results were expressed as mean ± SD, and the p value less than 0.05 was considered statistically significant.

## Results

### Tissue-specific expression patterns of bovine *ELOVL6* transcripts

**Fig 1:**
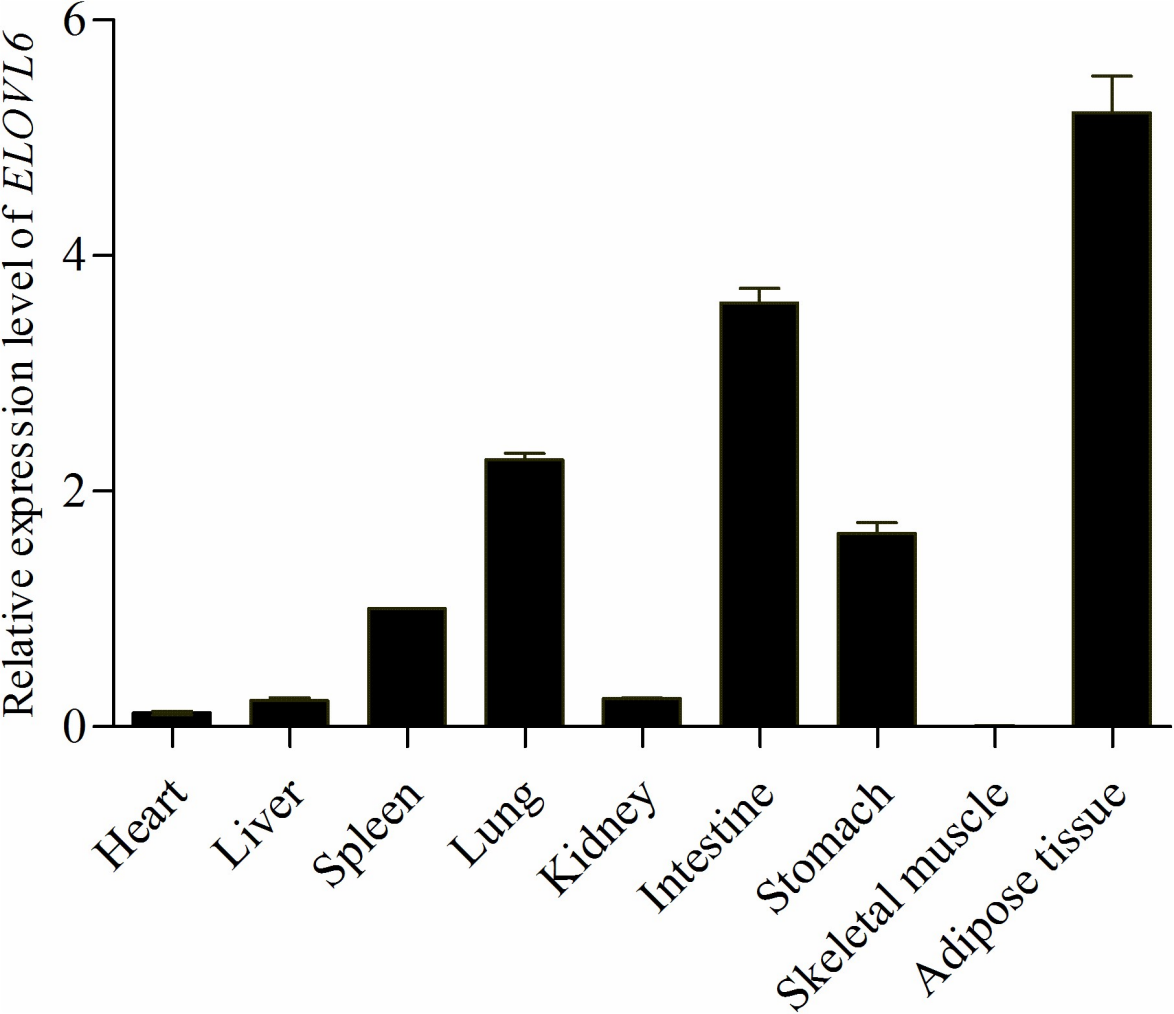
Tissue-specific expression patterns of bovine *ELOVL6* mRNAs. Each column represented the mean ± SD of three independent experiments which were performed in triplicate.

To enhance the understanding of the transcriptional regulation mechanism of bovine *ELOVL6* in various tissues of *Qin Chuan* cattle, we investigated the expression profiles of bovine *ELOVL6* in nine different tissues by qPCR. *ELOVL6* was expressed ubiquitously on bovine (Fig 1). Significantly high transcript levels were observed in adipose tissue and intestine. The expression of bovine *ELOVL6* in lung, stomach and spleen displayed moderate transcript levels, followed by kidney, liver, heart. Skeletal muscle had the lowest transcript level among the tissues investigated.

### Bioinformatics analyses of bovine *ELOVL6* promoter

**Fig 2:**
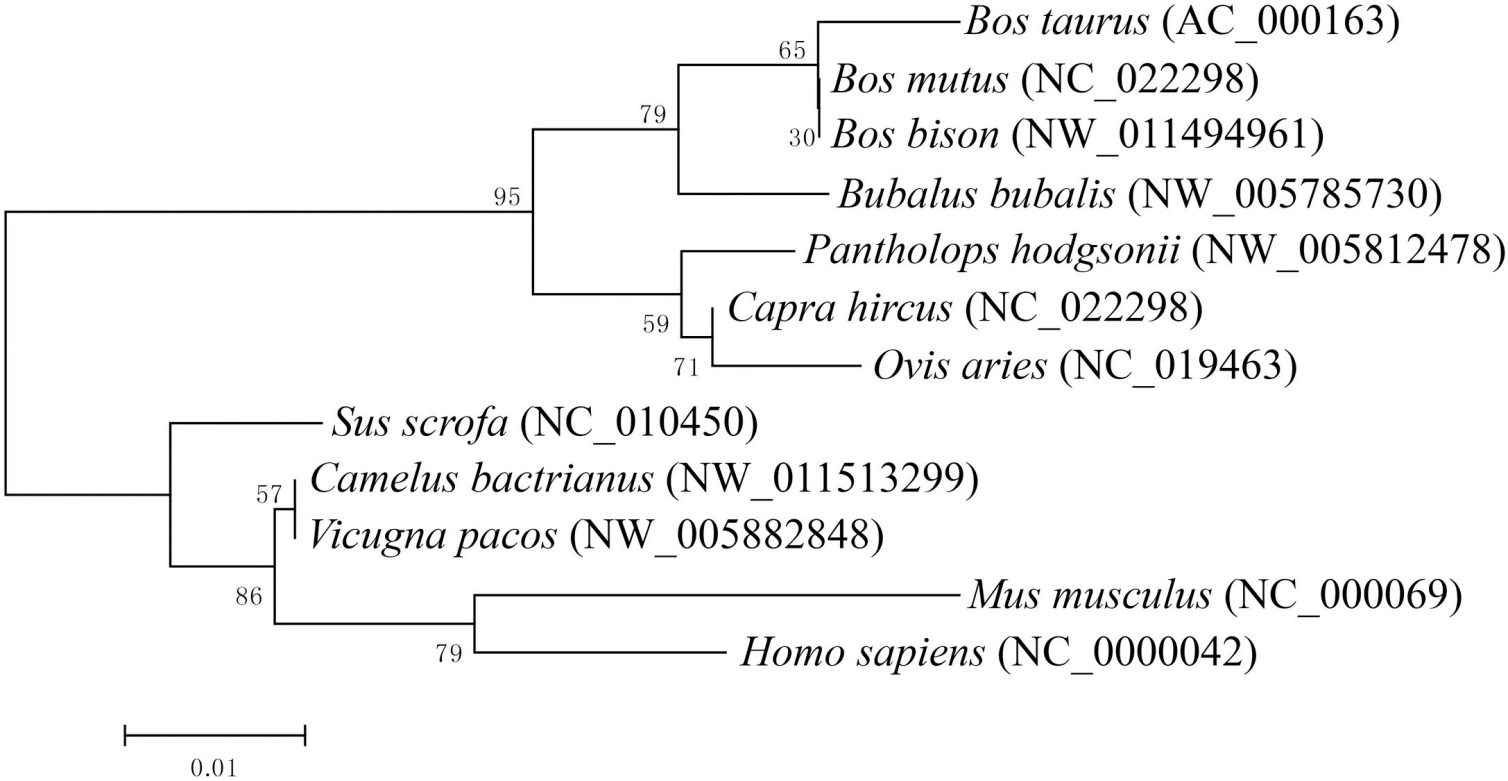
The phylogenetic analyses of bovine *ELOVL6* promoter. The phylogenetic relationship was analyzed by Neighbor Joining method (Mega program version 6.0) utilizing bovine *ELOVL6* promoter and homologous sequences from other mammal species. Bootstrap values were obtained from 1000 repetitions and illustrated as percentages at the nodes. The evolutionary distance of 0.01 nucleic acid substitutions per position was represented at the scale bars.

The 5’-flanking region of bovine *ELOVL6* promoter was amplified and was identical to the GenBank database (accession no. AC_000163). To understand potential evolutionary processes of bovine *ELOVL6* promoter among mammal species, a neighbor-joining phylogenetic tree was constructed by MEGA program (version 6.0). *Bos Taurus* has the close relationship with *Bos mutus* and *Bos bison* among *Bovidae* family. *Mus musculus* and *homo sapiens* display the greatest distance from *Bos taurus* (Fig 2).

**Fig 3:**
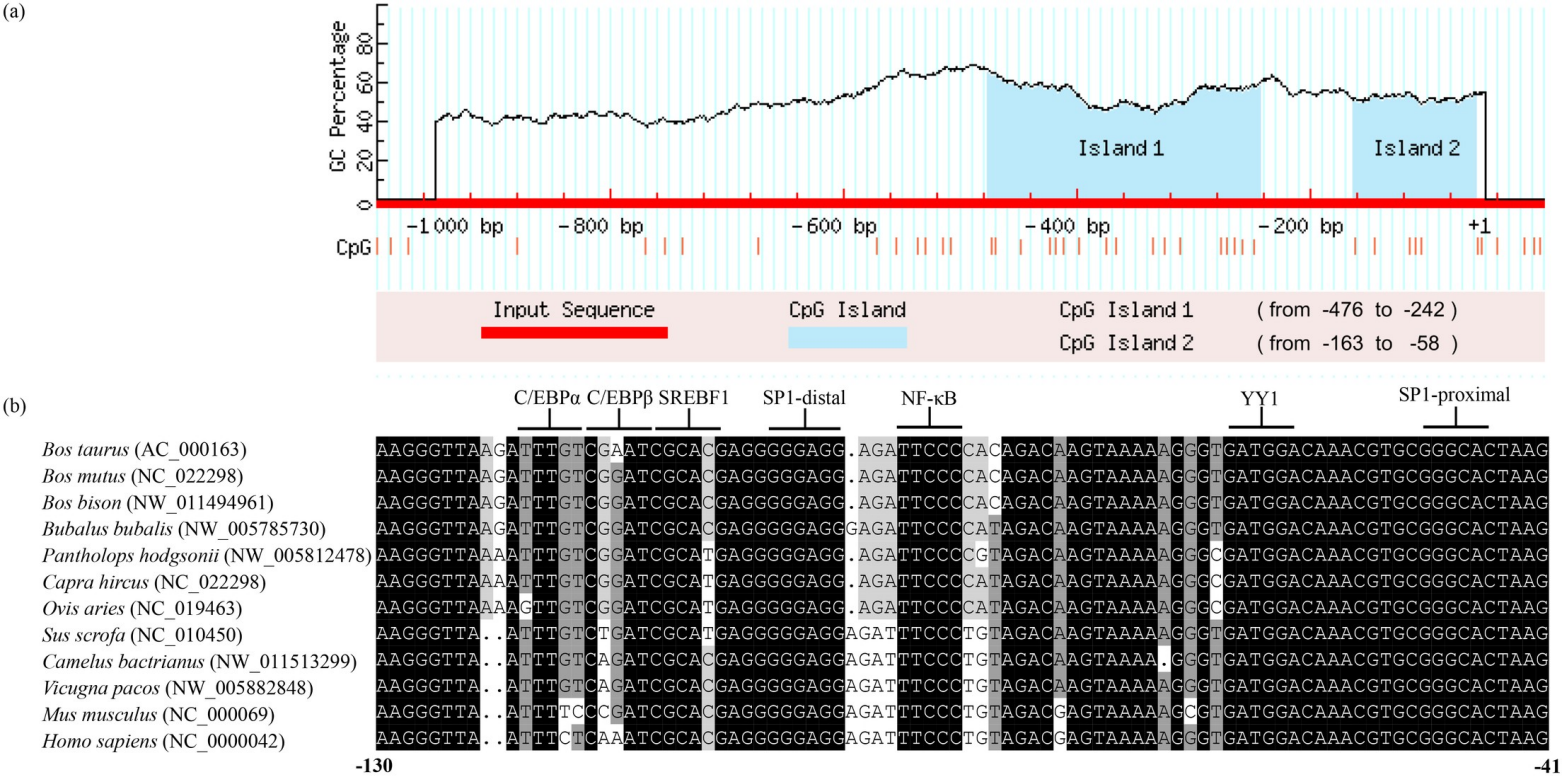
The prediction and analyses of CpG islands in bovine *ELOVL6* promoter. (**a**) The predicted CpG islands in the bovine *ELOVL6* promoter (+1 to -1000 bp). The red vertical illustrated the GC-rich regions. The blue shading regions indicated the predicated CpG islands; (**b**) Multiple sequence alignment of the second CpG island. Seven predicted transcription factors binding sites were underlined. Black shaded sequences indicated that the base pair was identical in all sequences of the alignment. Dark grey shadow indicated conserved substitutions and light grey shadow illustrated semi-conserved substitutions.

Utilizing the MatInspector program (Genomatix, USA), we analyzed the cloned promoter fragment consisting 980 bp upstream of the transcription start site (TSS). Interestingly, neither a TATA box nor a CAAT box was identified at the upstream of TSS. However, this region was found to be GC-rich. The online program MethPrimer revealed two predicted CpG islands within the promoter region of the bovine *ELOVL6* (Fig 3A). Conserved nucleic acids were identified in the proximal CpG island by multiple sequence alignment (Fig 3B). Subsequently, we analyzed the sequence between -130 bp to -41 bp, a total of seven transcription factor binding sites were predicted on both strands of the promoter core sequence.

### Identification of the core promoter region

**Fig 4:**
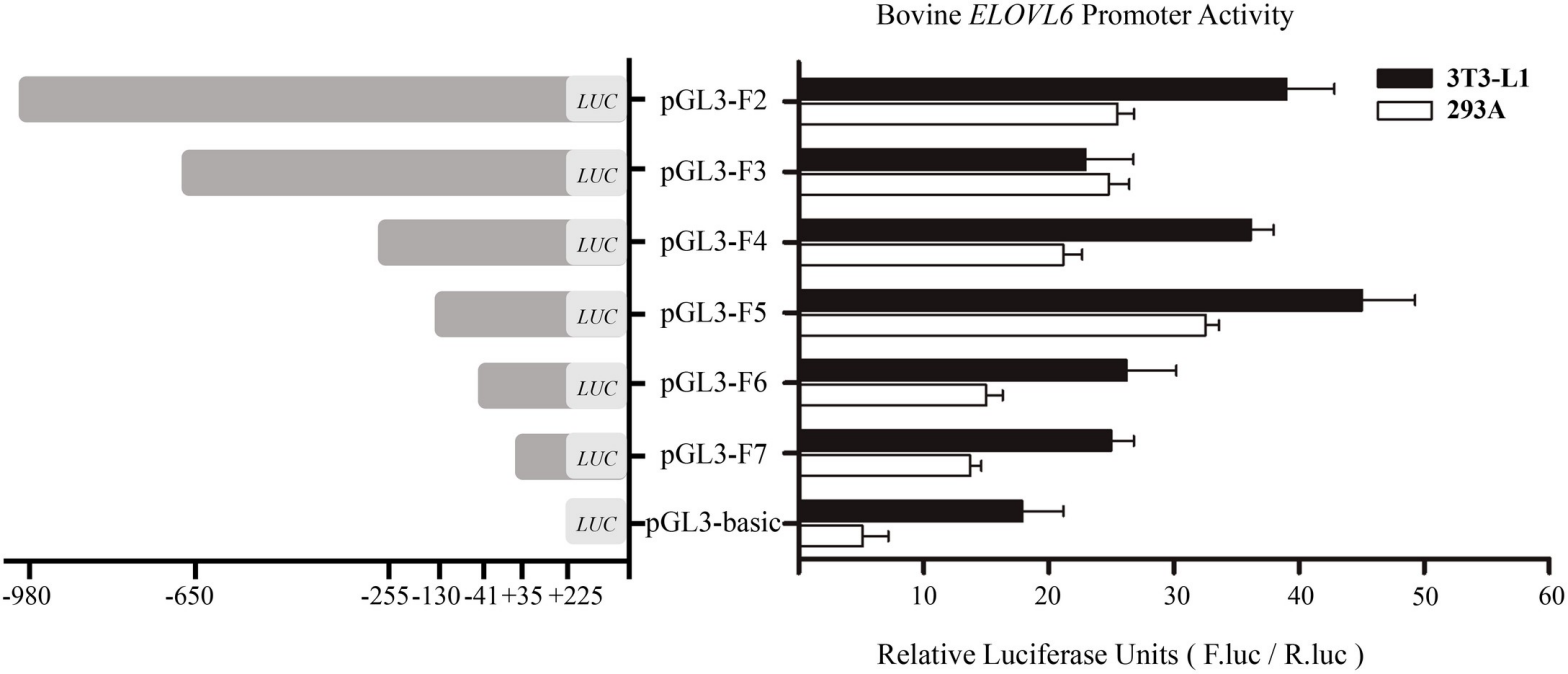
The structures and promoter activities of various truncation constructs of bovine *ELOVL6* promoter region. The promoter activities of 3T3-L1 cells (black bars) and 293A cells (white bars) were shown as the mean ± SD of the independent experiment performed in triplicate.

In order to narrow down the core region of bovine *ELOVL6* promoter, we constructed six pGL3 reporter plasmids (designated as F2 to F7), which contained unidirectional truncations (from 5’ to 3’) of bovine *ELOVL6* promoter, and then co-transfected with pRL-TK plasmid into 3T3-L1 and 293A cell lines for dual-luciferase reporter assay. In 3T3-L1 cell line, the dual-luciferase reporter assay showed that bovine *ELOVL6* promoter activity gave a “down-up-down” pattern along with the piecewise truncation (Fig 4). Significantly higher promoter activity was observed in 3T3-L1 cell line transfected with the constructs containing the region between -130 bp and -41 bp, which indicated that this fragment contained the core region of bovine *ELOVL6* promoter and probably harbored important transcriptional factor binding sites. Meanwhile, similar results were obtained in 293A cell line.

### Deletion of potential transcription factor binding sites

**Fig 5:**
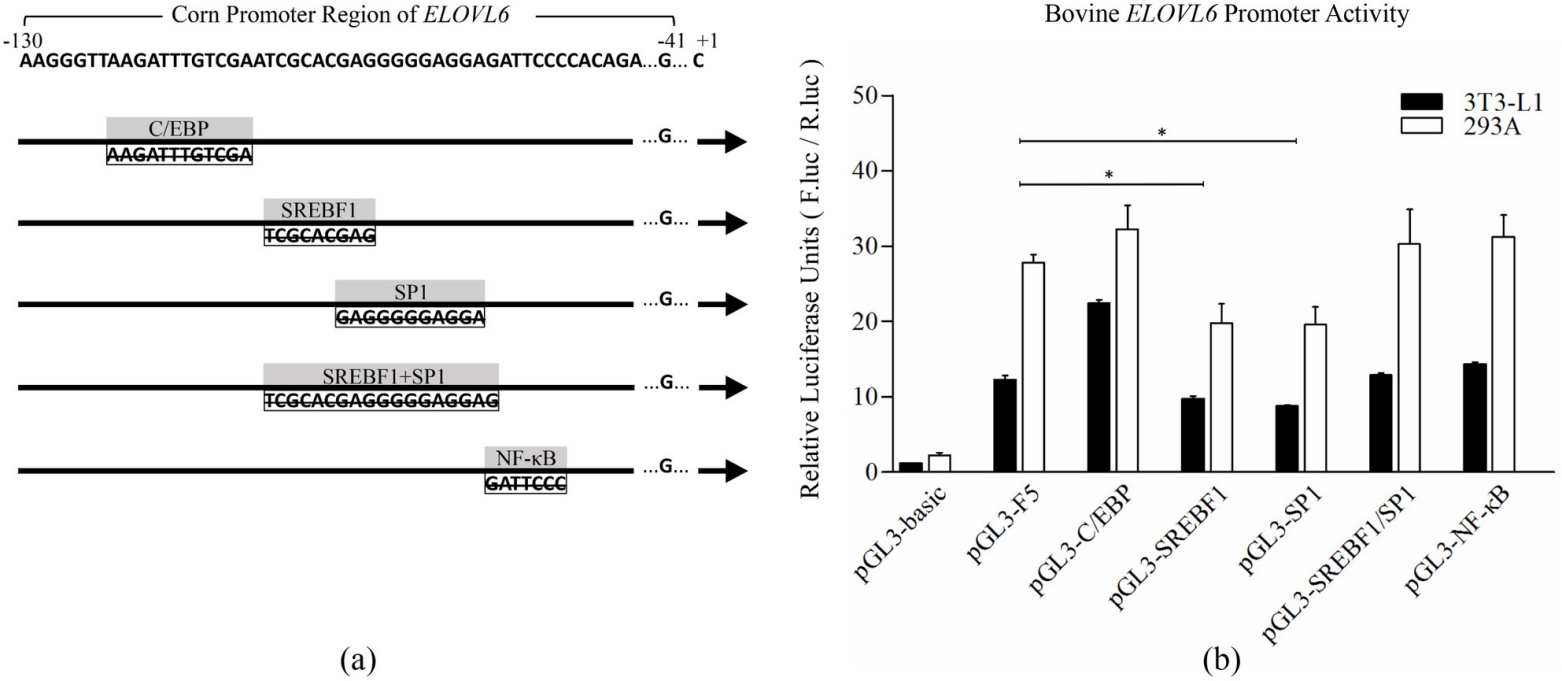
Luciferase activities of different deletion constructs. (**a**) Schematic represented various deletion constructs in the core region of bovine *ELOVL6* promoter. The transcription initiation site was designated as +1.; (**b**) Luciferase activities of different deletion constructs were indicated as mean ± SD of the independent experiment performed in triplicate.

The significant decrease of promoter activity in pGL3-F6 construct (-41/+225 bp) which was compared with pGL3-F5 construct (-131/+225 bp) may be attributed to the loss of cis elements contained within the deleted DNA region. To confirm the hypothesis, we chose four predicted transcription factor binding sites involved in fatty acids synthesis pathway to investigate their effects on bovine *ELOVL6* promoter activity. We performed the deletion mutation of potential transcription factor binding sites to identify the key regulatory elements, which was schematically represented in Fig 5A. After measured by dual-luciferase reporter assay, no significant change in promoter activity was found in the deletions of C/EBP and NF-κB binding sites, suggesting that C/EBP and NF-κB may not be dominant regulators for bovine *ELOVL6* promoter. In contrast, the respective deletion mutation of SREBF1 and SP1 binding sites dramatically decreased the promoter activities, which indicated SREBF1 and SP1 may be positive and dominant transcriptional regulators for bovine *ELOVL6* promoter (Fig 5B).

### Silence of *SREBF1* and *SP1* in bovine mammary epithelial cells (BMECs)

**Fig 6:**
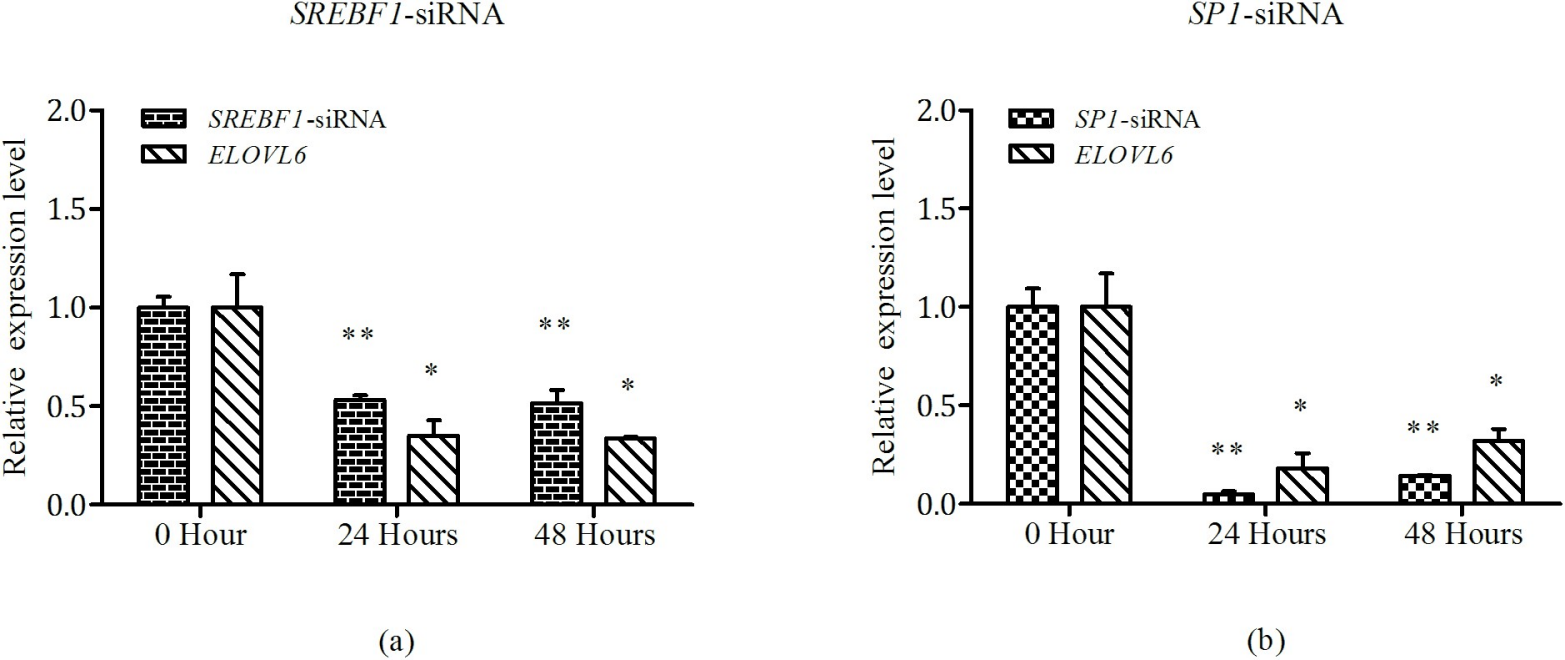
siRNA decreased the mRNA expression of *ELOVL6*. (**a**) The silencing of *SREBF1* affected the mRNA expression of *ELOVL6*; (**b**) The silencing of *SP1* affected the mRNA expression of *ELOVL6*. GAPDH was measured as the reference gene. Each column represented the mean ± SD of the independent experiment performed in triplicate. * p < 0.05; ** p < 0.01.

Based on estimates of cell volume, maximum intracellular concentration of siRNA is on the order of 5 pmol. After treatment with siRNA of *SREBF1* and *SP1* for 24 hours and 48 hours, respectively, both mRNA expression of *ELOVL6* were significantly decreased. Relative to the experission in 0 hour, silencing of *SREBF1* in 24 hours markedly decreased 65% expression of *ELOVL6* (Fig 6A). Meanwhile, the expression of *ELOVL6* in *SP1* silencing group were induced a 82% reduction compare with 0 hour group (Fig 6B). Similar results were obtained in 48 hours group. These results demonstrated that both SREBF1 and SP1 regulated the transcription of bovine *ELOVL6*.

## Discussion

VLCFAs are fatty acids with greater than 20 carbon atoms, which can be characteristically divided into saturated, monunsaturated and polyunsaturated fatty acids [10]. VLCFAs regulate a variety of cellular functions and improve the resistance of numerous diseases, for instance cardiovascular disease and metabolic syndrome [1]. The enzymes involved in the process of VLCFAs synthesis were regulated by dietary [27] and transcription factors [28]. A recent study has indicated that PUFAs synthesis is regulated more by substrate competition for existing enzymes than by an increase in their mRNA expression [29]. In this research, we attempted to improve the fatty acids profiles of beef and milk, and raise the content of VLCFA through increasing its precursor stearic acid. Numerous studies have demonstrated that stearic acid which is the precursor of VLCFA was specifically elongated from palmitic acid by ELOVL6 [8, 11, 13]. Understanding the transcriptional regulation of bovine *ELOVL6* resulted in ways towards guiding the production of high-quality beef and increasing the commercial value of cattle. Data derived from mouse has provided that the core region of mouse *ELOVL6* promoter contained an E-box and a SREBF1 binding site (SRE) [22]. Further works demonstrated that SREBF1 were the dominant transcription factors for mouse *ELOVL6* [13, 14]. The findings on human compared with murine revealed another transcriptional regulation pattern that ChREBP and SREBF1 synergistically activated human *ELOVL6* promoter [23]. ELOVL1, as a member of ELONGASE family, was regulated by mTOR through the activating the transcription factors SREBF1 and PPARγ in Cashmere goat [30]. The transcription factor type and its regulatory pattern were strongly determined by species and cell type [31]. We hypothesized that the transcriptional regulation of bovine *ELOVL6* would be different within patterns on mouse, human or goat.

In general, *ELOVL6* mRNAs are ubiquitously expressed but their ratios vary across different tissues. In the current study, we demonstrated that the expression profile of bovine *ELOVL6* slightly differ from that in human. In bovine, the transcript levels of *ELOVL6* in intestine was higher than that in lung, whereas opposite observation was found in human as previously described by Ohno [11]. However, similar expression patterns were observed in other tissues. The different expression patterns in intestine were probably owning to the high fat diets for fattening beef cattle, which may increase the expression of bovine *ELOVL6*. The small intestine is commonly thought of as a lipid storage organ, however, a recent research clarified a novel function of intestine that enterocytes stored the dietary fat in cytoplasmic lipid droplets (CLDs), when meals and diets containing large amounts of fat were consumed [32]. In order to alleviate the lipotoxicity to enterocytes induced by high concentrations of free fatty acids absorbed from high fat diets, enterocytes required high expression level of *ELOVL6* to elongate the palmitic acid into stearic acid for the storage in CLDs [33]. Since the limitation of samples collection, we were fail to investigate the tissue expression of bovine *ELOVL6* on cow.

We have identified that the core region (-131/-41 bp) of bovine *ELOVL6* promoter located at the second putative CpG island, harboring seven predicted TFBSs. The findings consisted with previous report that the DNA methylation of CpG island influenced the binding between transcription factor and its target DNA [34]. Among the seven predicted TFBSs, we chose four sites to verify the key transcription factors. C/EBP and SREBF1 play pivotal roles in regulating genes associated with fatty acid synthesis [35]. A recent study has been demonstrated that SP1 which belongs to Sp/Kruppel super family regulated gene related to fatty acids metabolism in goat [36]. NF-κB is recruited to many of the enhancer elements associated with the set of repressed-induced genes for inhibiting the reprogramming of fatty acid metabolism [37]. The site-directed deletion mutation assay in bovine *ELOVL6* promoter proved our hypothesis that both SREBF1 and SP1 can activate the transcription of bovine *ELOVL6* individually and exhibited equal effects on the promoter activities. For verification of results, we designed RNA interference of SREBF1 and SP1 in BMECs which was in line with previous findings.

Study on murine demonstrated that SREBFs including three isoforms (SREBF-1a, SREBF-1c and SREBF-2), are established as global lipid synthetic regulators [38]. However, only two isoforms, SREBF1 and SREBF2, have been identified on bovine. The research using RNA interference in bovine mammary epithelial cells revealed that SREBF1 plays an important role in integrated regulation of lipid synthesis through regulation of key enzymes, including ACC, FAS, FABP3, and SCD [39]. For providing the optimal transcriptional activation, SREBFs require additional coregulatory factors that, are limited to SP1, NF-Y or CREB to date [40]. Recent studies have shown that SP1 regulated the transcriptional activity of FAS and acyl-CoA synthetase long-chain family member 1 (ACSL1) which involved in fatty acids synthesis [41, 42]. SP1 regulates the transcription via two different mechanisms that SP1 can directly bind to the promoter and activate the transcription of adipogenesis genes, as well as which can indirectly enhance the expression of these genes by up-regulating SREBF1 [43, 44]. Taken together, these findings indicated transcription factors not work individually instead of cooperatively functioning as a comprehensive regulation network, integrating multiple signaling pathways at specific genomic regions. However, in our current study, we found simultaneous deletion of SREBF1 and SP1 binding sites reverted the promoter activity to a higher level than that of two single deletions but still lower than that of wild type, leaving a curious question for the regulation pattern in bovine *ELOVL6* promoter. We speculated that SREBF1 and SP1 synergistically regulated bovine *ELOVL6* promoter activity, which should be verified by our further works.

## Supporting information

S1 Table qPCR primers used in this work. (DOC)

S2 Table Primers of constructed dual-luciferase reporter plasmids used in this work. (DOC)

S3 Table Site-directed deletion mutation primers used in this work. (DOC)
