# Supplementary material for: Tissue expression profiles and transcriptional regulation of elongase of very long chain fatty acid 6 in bovine mammary epithelial cells
Source: PLoS One. 2017 Apr 17;12(4):e0175777. doi: 10.1371/journal.pone.0175777 (PMC5393602; doi:10.1371/journal.pone.0175777)
Supplement: S1 Table — (DOC) [file pone.0175777.s001.doc]

**S1 Table. qPCR primers used in this work.**

| Primers | | Direction | Primers Sequence(5`-3`) |
| --- | --- | --- | --- |
| Bos-GAPDH | | Forward | ATCATCTCTGCACCTTCTGCCGAT |
|  | | Reverse | TAAGTCCCTCCACGATGCCAAAGT |
| Bos-ELOVL6 | | Forward | GTGGTCGGCACCTAATG |
|  | | Reverse | AACCCTGGTCACAAACTG |
| Bos-SREBF1-RT | | Forward | CTGTCCACAAAAGCAAATCGC |
|  | | Reverse | CCACTTCCACCGCTGCTACTG |
| Bos-Sp1-RT | | Forward | GGAATACATGATGACCCAACAGG |
|  | Reverse | | AGCCCCTTCCTTCACTGTCTTTA |
