# Supplementary material for: Tissue expression profiles and transcriptional regulation of elongase of very long chain fatty acid 6 in bovine mammary epithelial cells
Source: PLoS One. 2017 Apr 17;12(4):e0175777. doi: 10.1371/journal.pone.0175777 (PMC5393602; doi:10.1371/journal.pone.0175777)
Supplement: S2 Table — (DOC) [file pone.0175777.s002.doc]

**S2 Table. Primers of constructed dual-luciferase reporter plasmids used in this work.**

| Primers | Primers Sequence(5`-3`) | Position | Usage | Size | | Enzyme |
| --- | --- | --- | --- | --- | --- | --- |
| P-F2 | CG***ACGCGT***TATTAGACGCCTGGATGT | -980 to +224 | Amplification F2 with P-R1 | | 1222bp | *Mlu*I |
| P-F3 | CG***ACGCGT***GCTGCTGCACTACCTCTA | -650 to +224 | Amplification F3 with P-R1 | | 892bp | *Mlu*I |
| P-F4 | CG***ACGCGT***GACAGGGCGTTGTAGATG | -255 to +224 | Amplification F4 with P-R1 | | 497bp | *Mlu*I |
| P-F5 | CG***ACGCGT***AAGGGTTAAGATTTGTCG | -130 to +224 | Amplification F5 with P-R1 | | 372bp | *Mlu*I |
| P-F6 | CG***ACGCGT***ACCGCAAGGCATTCATTT | -41 to +224 | Amplification F6 with P-R1 | | 283bp | *Mlu*I |
| P-F7 | CG***ACGCGT***GCGGAGGCACAGGCAATG | +35 to +224 | Amplification F7 with P-R1 | | 207bp | *Mlu*I |
| P-R1 | CCG***CTCGAG***ATCTTCGGAGTCGCTACG |  | Common reverse primer | |  | *Xho*I |
