# Supplementary material for: Tissue expression profiles and transcriptional regulation of elongase of very long chain fatty acid 6 in bovine mammary epithelial cells
Source: PLoS One. 2017 Apr 17;12(4):e0175777. doi: 10.1371/journal.pone.0175777 (PMC5393602; doi:10.1371/journal.pone.0175777)
Supplement: S3 Table — (DOC) [file pone.0175777.s003.doc]

**S3 Table. Site-directed deletion mutation primers used in this work.**

| Primers | | Direction | | Primers Sequence(5`-3`) | |
| --- | --- | --- | --- | --- | --- |
| C/EBP del | Forward | | CTTCGATAGGTACCGAGCTCTTACGCGTAAGGGTTATCGCACGAGGGGGAGGAG | |  |
|  | Reverse | | GAAGCTATCCATGGCTCGAGAATGCGCATTCCCAATAGCG | |  |
| SRE del | Forward | | CGAGCTCTTACGCGTAAGGGTTAAGATTTGTCGAAGGGGAGGAGATTCCCCACAG | |  |
|  | Reverse | | GCTCGAGAATGCGCATTCCCAATTCTAAACAGCTTCCC | |  |
| Sp1 del | Forward | | TTACGCGTAAGGGTTAAGATTTGTCGAATCGCACGATTCCCCACAGACAAGTAAAAAG | |  |
|  | Reverse | | AATGCGCATTCCCAA TTCTAAACAGCTTAGCGTGCTAAGG | |  |
| SRE/Sp1 del | Forward | | CGAGCTCTTACGCGTAAGGGTTAAGATTTGTCGAAATTCCCCACAGACAAGTAAAAAG | |  |
|  | Reverse | | GCTCGAGAATGCGCATTCCCAATTCTAAACAGCTTTAAGG | |  |
| NF-κB del | Forward | | GAGGACACACAGACAAG | |  |
|  | Reverse | | CTTGTCTGTGTGTCCTC | |  |
